# *HYAL1* and *HYAL2* Inhibit Tumour Growth *In Vivo* but Not *In Vitro*

**DOI:** 10.1371/journal.pone.0003031

**Published:** 2008-08-22

**Authors:** Fuli Wang, Elvira V. Grigorieva, Jingfeng Li, Vera N. Senchenko, Tatiana V. Pavlova, Ekaterina A. Anedchenko, Anna V. Kudryavtseva, Alexander Tsimanis, Debora Angeloni, Michael I. Lerman, Vladimir I. Kashuba, George Klein, Eugene R. Zabarovsky

**Affiliations:** 1 Microbiology and Tumour Biology Center, Karolinska Institute, Stockholm, Sweden; 2 Institute of Molecular Biology and Biophysics, SD RAMS, Novosibirsk, Russia; 3 Engelhardt Institute of Molecular Biology, Russian Acad. Sciences, Moscow, Russia; 4 Bioactivity Ltd., Rehovot, Israel; 5 Cancer-Causing Genes Section, Laboratory of Immunobiology, Center for Cancer Research, National Cancer Institute, Frederick, Maryland, United States of America; 6 Scuola Superiore Sant'Anna and Institute of Clinical Physiology – CNR, Pisa, Italy; 7 Institute of Molecular Biology and Genetics, Ukrainian Academy of Sciences, Kiev, Ukraine; Ordway Research Institute, United States of America

## Abstract

**Background:**

We identified two 3p21.3 regions (LUCA and AP20) as most frequently affected in lung, breast and other carcinomas and reported their fine physical and gene maps. It is becoming increasingly clear that each of these two regions contains several TSGs. Until now TSGs which were isolated from AP20 and LUCA regions (e.g.*G21/NPRL2, RASSF1A, RASSF1C, SEMA3B, SEMA3F, RBSP3*) were shown to inhibit tumour cell growth both *in vitro* and *in vivo*.

**Methodology/Principal Findings:**

The effect of expression *HYAL1* and *HYAL2* was studied by colony formation inhibition, growth curve and cell proliferation tests *in vitro* and tumour growth assay *in vivo*. Very modest growth inhibition was detected *in vitro* in U2020 lung and KRC/Y renal carcinoma cell lines. In the *in vivo* experiment stably transfected KRC/Y cells expressing *HYAL1* or *HYAL2* were inoculated into SCID mice (10 and 12 mice respectively). Tumours grew in eight mice inoculated with *HYAL1*. Ectopic *HYAL1* was deleted in all of them. *HYAL2* was inoculated into 12 mice and only four tumours were obtained. In 3 of them the gene was deleted. In one tumour it was present but not expressed. As expected for tumour suppressor genes *HYAL1* and *HYAL2* were down-expressed in 15 fresh lung squamous cell carcinomas (100%) and clear cell RCC tumours (60–67%).

**Conclusions/Significance:**

The results suggest that the expression of either gene has led to inhibition of tumour growth *in vivo* without noticeable effect on growth *in vitro*. *HYAL1* and *HYAL2* thus differ in this aspect from other tumour suppressors like P53 or *RASSF1A* that inhibit growth both *in vitro* and *in vivo*. Targeting the microenvironment of cancer cells is one of the most promising venues of cancer therapeutics. As major hyaluronidases in human cells, *HYAL1* and *HYAL2* may control intercellular interactions and microenvironment of tumour cells providing excellent targets for cancer treatment.

## Introduction

We have performed a deletion survey of 3p on more than 400 lung, renal, breast, cervical and ovarian carcinomas using a defined set of markers, combining conventional LOH (loss of heterozygosity) with quantitative real-time PCR (qPCR), comparative genomic and NotI microarrays hybridisations [1]–[5]. We identified two most frequently affected 3p21.3 regions, LUCA at the centromeric and AP20 at the telomeric border of 3p21.3. Aberrations of either region were detected in more than 90% of the studied tumours. Homozygous deletions (HD) were detected in 10%–18% of all tumours at both the LUCA and AP20 sites [4], [5]. The frequent chromosome losses in these regions suggest that they harbor multiple tumour suppressor genes (TSG) [6]–[8]. It was suggested that aberrations in both the LUCA and AP20 region could be functionally linked [5], [9], [10].

The definition of a TSG is based on the demonstration of its regular inactivation by mutation or epigenetic silencing in tumour samples. It is also important to obtain supportive evidence from functional studies. We have previously found (in collaboration with Stefan Imreh et al.) non-random losses of human 3p21-p22 fragments from mouse-human microcell hybrids following progressive growth in SCID mice [11]. In order to test whether a known suppressor gene, RB, would behave in a similar way, wild type and mutated RB genes were introduced into the pETE (Elimination Test Episomal) vector that permitted the expression of the gene in the absence but not in the presence of tetracycline. The expression of the gene could be modulated by tetracycline both *in vivo* and *in vitro*. When the transfectants were passaged as tumours in immunodeficient SCID mice, the wild type RB gene was deleted or functionally inactivated already after the first passage in all 20 tumours tested. In contrast, a non-functional mutant RB gene was maintained in all 10 tumours studied. In similar experiments with wt P53, the exogenous P53 gene was maintained and expressed in all 6 tumours tested, but in a mutated form. On the basis of these experiments we have developed the gene inactivation test (GIT) for a functional definition of TSG. It is based on the comparison of cell growth *in vitro* and tumour growth *in vivo* when the gene is/is not expressed. The main idea of the test is that a gene inhibiting growth of tumour cells should be inactivated in growing tumours by genetic or epigenetic mechanisms.

Using GIT and growth analysis under cultural conditions we have shown that *FUS1*, *SEMA3B*, *G21/NPRL2*, *RASSF1A*, *RASSF1C, RBSP3* (genes from AP20 and LUCA regions) and other TSGs inhibit tumour cell growth both *in vitro* and *in vivo* [7], [9], [11]–[15].

However other genes from 3p21.3 (e.g. *TCEA1*, *MLH1*, *RHOA*, *3PK*, *PL6*, *101F6*, *BLU*, *TGFBR2*) did not show any effect in the tested cell lines [11].

Here we describe the functional analysis of two additional genes from the 3p21.3 LUCA region, hyaluronidases -1 and -2 (*HYAL1* and *HYAL2*).

The *HYAL1* gene (hyaluronoglucosaminidase 1) is located in the LUCA region. It contains 6 exons producing a 2.6 kb mRNA (coding for 436 aa protein). It is well expressed in all analysed normal human tissues including lung and kidney. It was not expressed in 18 out of 20 lung cancer cell lines [6].

The *HYAL2* (hyaluronoglucosaminidase 2, located in LUCA region) contains 4 exons producing a 2 kb mRNA (that encode 473 aa). It is well expressed in all analysed human tissues including lung, kidney and many lung cancer cell lines. The protein is attached to the membrane by the glycosylphosphatidyl-inositol-anchor (GPI-anchor) [16]. The *HYAL2* protein was identified as a receptor for the sheep lung cancer retrovirus, JSRV, and a sequestration mechanism inactivating *HYAL2* protein was demonstrated. The *env* gene of JSRV was shown to transform human bronchial epithelial cells *in vitro* and sequesters the *HYAL2* protein. The absence of *HYAL2* (mediated either by a putative virus or mutational inactivation) leads to ligand-independent activation of the *RON* receptor tyrosine kinase and its downstream *AKT* and *MAPK* signaling pathways [17].

These two genes contribute to intracellular and extracellular catabolism of hyaluronic acid (HA) in a CD44-dependent manner [18]. HA has a great number of biological functions: it mediates cell-cell and cell-matrix interactions and plays an important role in cell migration, tumour growth and progression.

Thus analysis of *HYAL1* and *HYAL2* can have a great importance not only for better understanding of HA catabolism and human carcinogenesis but could provide therapeutic targets for cancer treatment. In this study we have found that the expression of either gene suppressed tumour growth *in vivo* but not *in vitro*. These findings are consistent with earlier somatic hybrid studies by Henry Harris, George Klein and Francis Wiener, showing that somatic hybridisation of normal and malignant cells can suppress tumorigenicity *in vivo* but not cell growth *in vitro* [19], [20]. The mechanisms of the *in vivo* suppressive effects have not been clarified, but at least part of them may have acted at the level of tumour-host interactions. The finding of a similar phenomenon with the two genes involved in the present study is a step towards the analysis of this important phenomenon.

## Results

### Colony formation inhibition by HYAL1 and HYAL2 in vitro

**Figure 1:**
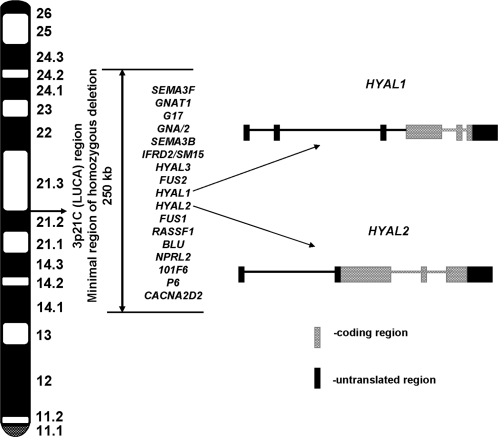
Schematic map of the LUCA region picturing the *HYAL1* and *HYAL2* genes.

In this study we performed initial functional analysis of *HYAL1* and *HYAL2*. These genes are located in the minimally deleted region of LUCA (Figure 1). The work was a continuation of our previous study to characterize different genes, located in the same region, e.g. *RASSF1A* [13], *RASSF1C* [14] and *G21/NPRL2* [15]. These studies showed that these genes have strong growth inhibiting activity both *in vitro* and *in vivo*.

Expression of *HYAL1* and *HYAL2* was almost undetectable in the KRC/Y renal and U2020 lung carcinoma lines (data not shown), which were used in our growth suppression experiments for testing *RASSF1A* and *G21/NPRL2* [13]–[15]. As both *HYAL1* and *HYAL2* are well expressed in normal kidney and lung (see for example http://www.genecards.org/index.shtml) we have chosen KRC/Y and U2020 for our study.

**Figure 2:**
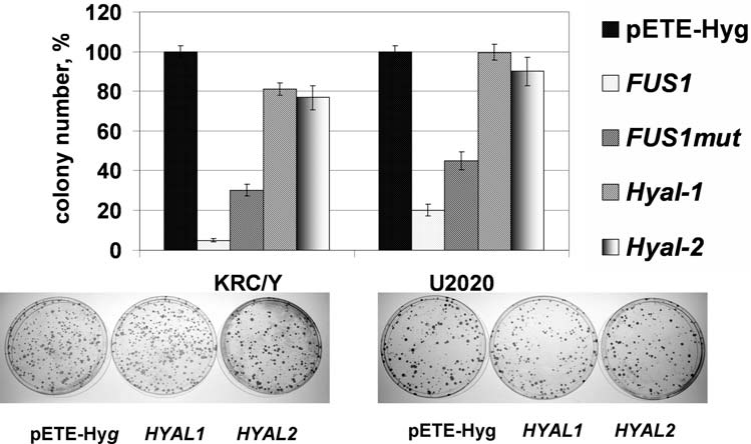
Effect of *HYAL1* and *HYAL2* transgenes on colony formation efficiency in KRC/Y and U2020 cells compared to empty pETE vector (negative control) and wild type or mutated *FUS1* transgenes. Graphical representation summarizing three independent experiments and photographic images of Petri dishes stained with methylene blue. Values are the mean ±s.d. of three separate experiments each calculated from triplicate plates.

*HYAL1* and *HYAL2* were cloned into the episomal tetracycline-regulated pETE-Hyg vector [21] and used for transfection of the KRC/Y and U2020 cells (for colony formation experiments, Figure 2). The empty pETE vector was used as a negative control. *HYAL1* and *HYAL2* showed almost no inhibition of colony formation. When *HYAL1* and *HYAL2* were expressed the cloning efficiency was 77–100%, compared to the empty vector. As a positive control we used *FUS1* gene, a strong TSG cloned in pETE [7]. Colony formation efficiency of the KRC/Y cells expressing *FUS1* was less than 5% and for U2020 cells it was less than 25% compared to controls.

**Figure 3:**
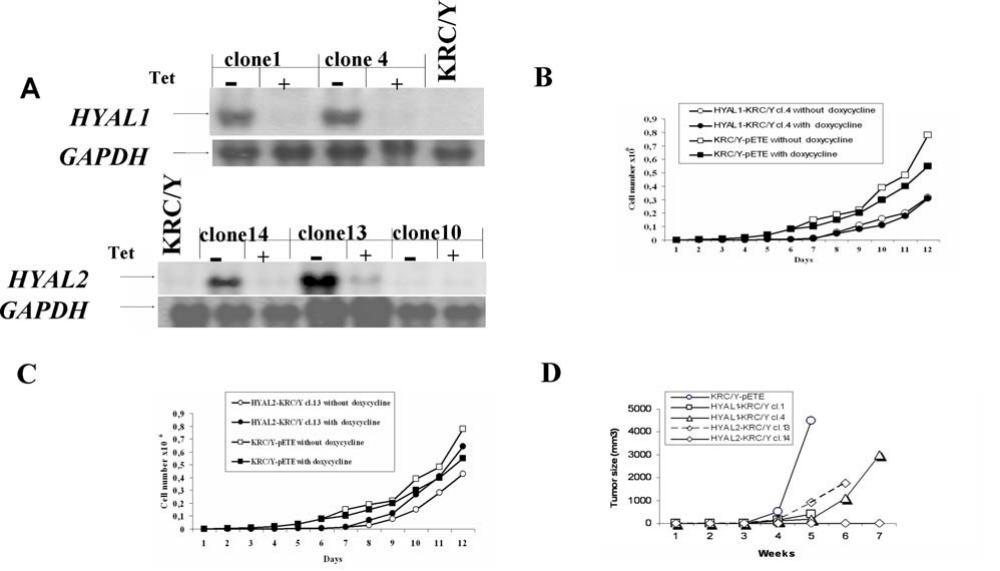
Analysis of *HYAL1* and *HYAL2* stably transformed KRC/Y clones. Northern analysis (A) of tetracycline regulated clones. (+), tetracycline (Tet) or doxycycline is present, gene is OFF. (−), tetracycline or doxycycline is absent, gene is ON. Growth inhibition of KRC/Y cells with *HYAL1* (B) and *HYAL2* (C) transgenes *in vitro*. Tumour growth inhibition of KRC/Y cells by *HYAL1* and *HYAL2 in vivo* in SCID mice (D). Mice were drinking water with tetracycline (+Tet, gene is OFF) or without (−Tet, gene is ON) but for simplicity curves are shown only for mice when genes were ON (no tetracycline).

We performed also growth curve inhibition experiments using KRC/Y cells stably transformed with *HYAL1* and *HYAL2* transgenes. The pETE-HYAL1 and pETE-HYAL2 vectors carrying wild type alleles of the tested genes were transfected into KRC/Y expressing tTA and clonal cell lines were selected. Expression of the transgenes was tested by Northern hybridization. Two of the best tetracycline regulated clones for each gene were then used in further experiments (see for example Figure 3A). For *HYAL1* clones 1 and 4 were selected (HYAL1-KRC/Ycl.1 and HYAL1-KRC/Ycl.4) and for *HYAL2* clones 13 and 14 (HYAL2-KRC/Ycl.13 and HYAL2-KRC/Ycl.14). Growth curves for HYAL1-KRC/Ycl.4 and HYAL2-KRC/Ycl.13 are shown in Figure 3B and C. Only modest growth inhibition was seen like in the colony assays. Two other clones showed similar results.

**Figure 4:**
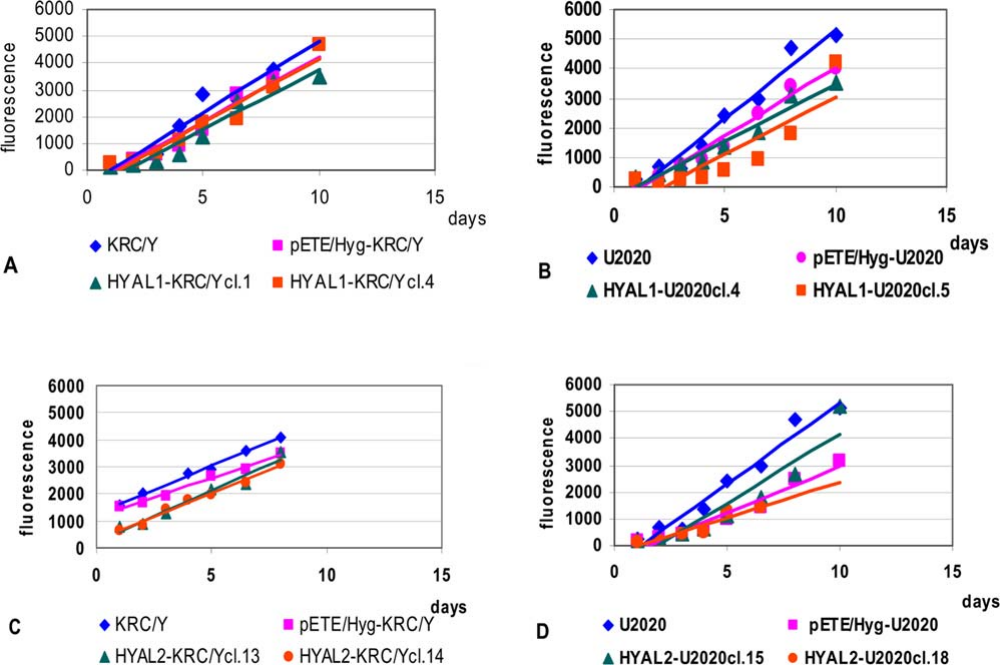
CyQUANT Cell Proliferation Assay. Effect of expression of *HYAL1* transgene in KRC/Y (A) and in U2020 (B) cells. The same is shown for *HYAL2* (KRC/Y in C and U2020 in D). Experiments were done in triplicates in the absence of doxycycline. The same experiments were done in the presence of doxycycline and showed similar results (data not shown). Plotted data points represent averages of triplicate samples, the plotted line is a linear regression fit of all data points. The assay is designed to produce a linear analytical response from at least 100–20,000 cells per well in most cell lines.

In the same way we selected U2020 cell clones expressing *HYAL1* (HYAL1-U2020cl.4 and HYAL1-U2020cl.5) and *HYAL2* (HYAL2-U2020cl.15 and HYAL2-U2020cl.18). All eight clones expressing either *HYAL1* or *HYAL2* in KRC/Y and U2020 cells were tested with CyQUANT NF Cell Proliferation Assay based on measurement of cellular DNA content via fluorescent dye binding (see Materials and Methods). Figure 4 shows that neither *HYAL1* nor *HYAL2* inhibited KRC/Y or U2020 cells to a significant extent.

### Gene inactivation test with HYAL1 and HYAL2 in SCID mice

We investigated *HYAL1* and *HYAL2* using the gene inactivation test (GIT) as described by Li *et al.*, 1999 [22] and Protopopov *et al*., 2002 [21]. The test mimics the inactivation of TSGs during tumour growth *in vivo*. Clonal cell lines expressing transgenes were inoculated into SCID mice and the expression of the transgene was controlled by tetracycline administered *ad libitum* in the drinking water. In this setting TSGs suppressed tumour formation in SCID mice, unless they were eliminated or mutated. All grown tumours were analysed for the presence and expression of the transgene.

KRC/Y derived *HYAL1* and *HYAL2* cell clones were inoculated into 22 SCID mice (6 mice/HYAL1-KRC/Ycl.1; 4 mice/HYAL1-KRC/Ycl.4; 8 mice/HYAL2-KRC/Ycl.13 and 4 mice/HYAL2-KRC/Ycl.14). For each SCID mouse, 5×10^6^ cells were inoculated (one inoculation per mouse). In control mice, KRC/Y cells transfected with empty pETE vector were used (twelve SCID mice were injected). Half of the SCID mice were then given drinking water containing 1mg/ml tetracycline. Results of these experiments are shown in Figure 3D (to make Figure more clear only curves for mice drinking water without tetracycline are shown). In many cases no tumour growth was observed at all (see Table 1). Strong inhibition of tumour growth was observed for all clones expressing *HYAL1* or *HYAL2*. All grown 12 tumours (T1–T12) were explanted and tested for the presence of pETE-HYAL1 and pETE-HYAL2 constructs by PCR. Transgenes were detected only in one tumour (*HYAL2*) and in eleven tumours it was deleted. In tumour T10 where the *HYAL2* gene was present by PCR, there was no detectable expression by Northern (data not shown).

**Table 1:** Growth of tumours in SCID mice.

Genes	Clone	Tetracycline	Tumours	PCR	Northern
*HYAL1*	clone1	-	T1	-	
		+	T2	-	
		-	No tumour		
		+	No tumour		
		-	T3	-	
		+	T4	-	
	clone4	-	T5	-	
		+	T6	-	
		-	T7	-	
		+	T8	-	
*HYAL2*	clone13	-	T9	-	
		+	T10	+	NO EXPR
		-	No tumour		
		+	No tumour		
		-	T11	-	
		+	T12	-	
		-	No tumour		
		+	No tumour		
	clone14	-	No tumour		
		+	No tumour		
		-	No tumour		
		+	No tumour		

### Expression of HYAL1 and HYAL2 genes is significantly decreased in lung and kidney cancer samples

As our data suggested that both *HYAL1* and *HYAL2* induce growth inhibition of tumours in SCID mice we tested expression of these genes in lung and renal tumours using qPCR (see Materials and Methods).

**Figure 5:**
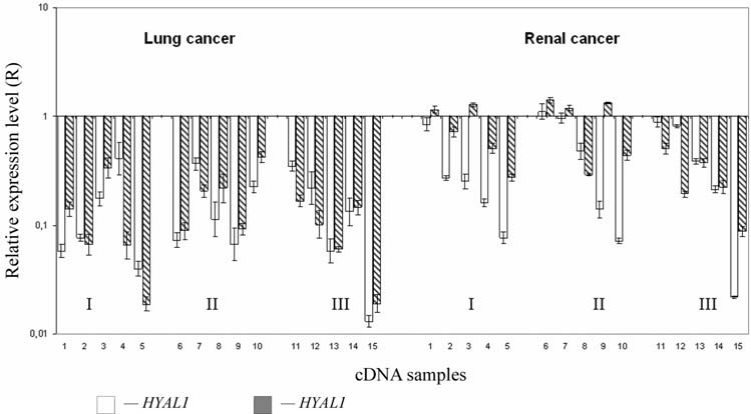
QPCR mRNA expression profile of *HYAL1* and *HYAL2* in SCC and RCC biopsies. The Y axis indicates the values of relative expression level of target genes in log_10_ scale in tumour samples relative to control normal samples normalized to the reference gene *GAPDH.* The X axis shows the cDNA samples isolated from tumours at different stages (I–III). Open bars show HYAL1 and hatched bars HYAL2 expression.

Fifteen SCC and fifteen RCC tumours were studied. We assumed that expression fell down if it was at least 2-fold lower than in matched control samples. Expression of both genes was drastically decreased in all lung samples (15/15, P<0.02, Figure 5). Average decrease level for *HYAL1* was 7.8 (from 2.5 up to 77) and for *HYAL2* 8.8 (2.5–53) times . In seven cases expression of *HYAL1* fell down more than 10-fold. For *HYAL2* eight such cases were found. In six of fifteen lung biopsies we found more than 10-fold decrease of both *HYAL1* and *HYAL2* expression. In RCC samples expression of *HYAL1* declined in 10 of 15 cases (67%, P<0.02) and average decrease was 6.5 times (from 2 to 46). For *HYAL2* reduced expression was detected in 9 of 15 samples (60%, P<0.02) and average decrease was 4.9 times (2–11.4). Only in three RCC samples expression of *HYAL1* decreased more than 10-fold and in one case we detected similar strong decline of *HYAL2* expression. No statistically significant difference in cases with different tumour stages was observed both in SCC and RCC cases.

## Discussion

In this work we describe functional characterization of *HYAL1* and *HYAL2* genes. Using the GIT test we previously tested altogether 15 genes from 3p21.3 LUCA and AP20 regions. Nine genes (*TCEA1*, *MLH1, VILL*, *RHOA*, *3PK*, *PL6*, *101F6*, *BLU*, *TGFBR2*) did not show any effect in the tested cell lines. Six genes (*RBSP3*, *NPRL2/G21*, *RASSF1A*, *RASSF1C*, *SEMA3B*, *SEMA3F*) had strong growth inhibitory activity, both *in vitro* and *in vivo*.

For example, *NPRL2/G21* demonstrated growth inhibition *in vitro* in small cell lung cancer cell line U2020 and renal carcinoma cell line KRC/Y cells almost 90%. *RASSF1A* gene showed the same results. In SCID mice both genes either did not permit tumour formation or in growing tumours they were inactivated (for *NPRL2* it was 10 mice and for *RASSF1A* it was 8 mice).

In contrast, neither *HYAL1* nor *HYAL2* showed significant growth inhibition in colony formation and growth curve assays *in vitro* (see Figures 2–3). CyQUANT NF Cell Proliferation Assay (Figure 4) also demonstrated that neither *HYAL1* nor *HYAL2* inhibited KRC/Y or U2020 cells proliferation. Importantly, pETE vectors expressed mRNA of inserted genes in quantity comparable to physiologically normal levels [14]. However in SCID mice both genes had a very strong inhibiting effect: no tumours grew in 10 mice and in 12 mice where tumours grew the genes were inactivated (deletion or loss of expression, see for example Figure 3D and Table 1). In our SCID mice experiments *HYAL1* and *HYAL2* genes were inactivated (or tumours didn't grow) even when the genes were repressed (water with tetracycline). However we have already shown that gene expression leakage *in vivo* is stronger than *in vivo*. Moreover it is known that tetracycline is a weaker inhibitor of expression compared to doxycycline in tTA system [9], [14], [15].

Thus, results clearly showed that the expression of either gene has led to the inhibition of tumour growth *in vivo*, most likely by influencing some interaction between the tumour cells and the host, but without directly affecting tumour cell growth *in vitro*. *HYAL1* and *HYAL2* thus differ in this aspect from other tumour suppressors like P53 or *RASSF1A* that inhibit growth both *in vitro* and *in vivo*. Since the products of both genes have a potential to influence intercellular interactions, their impairment may inhibit microenvironmental controls that normally protect the host from tumour growth.

*HYAL1* and *HYAL2* are the major mammalian hyaluronidases in somatic tissues. They may act in concert to degrade high molecular weight hyaluronan (HA, a negatively charged, high molecular weight glycosaminoglycan) to the tetrasaccharide level [23]. *HYAL2* hydrolyses high molecular weight hyaluronic acid (or hyaluronate or hyaluronan) to produce an intermediate-sized product which is further hydrolysed by *HYAL1* to give small oligosaccharides. Hyaluronan is claimed to be involved in tumour invasion and metastatic spread. The levels of HA surrounding tumour cells are often correlated with tumour aggressiveness and poor outcome [24].

Overproduction of HA enhances anchorage-independent tumour cell growth [25], [26]. Loss of hyaluronidase activity, permitting accumulation of HA, may be one of several steps required by cells in the multi-step process of carcinogenesis [23].

In contrast to the tumour suppressor function of *HYAL1* and *HYAL2* in these studies, no frequent inactivating mutations were identified so far in these genes. Nevertheless it was reported that *HYAL1* was inactivated in six of seven head and neck squamous cell carcinoma lines by illegitimate splicing [23], [27]. We found that the *HYAL2* promoter was methylated in more than 50% of non- small cell lung cancer (NSCLC) cases with decreased expression of the *HYAL2* (V. Senchenko, personal communication).

Suppressor activity of *HYAL1* and *HYAL2* was investigated in the study of Ji *et al.* [28]. The results showed that *HYAL1* and *HYAL2* did not significantly inhibit tumour cells growth neither *in vitro* nor *in vivo*. However in this work it was demonstrated that *HYAL2* inhibited experimental lung metastases in *nu/nu* mice. It is difficult to compare this work with our study. They used non-small cell lung cancer cell lines and recombinant adenoviral vectors. Completely different methods were used to measure suppressor activity. Despite these differences main results are similar to our work: *HYAL1* and *HYAL2* do not have strong growth inhibiting activity *in vitro* and *HYAL2* displayed some tumour suppressor activity *in vivo*.

Since we demonstrated tumour suppressor function of *HYAL1* and *HYAL2* in SCID mice, we suggested that in primary renal and lung cancers expression of these genes might also be distorted. mRNA quantification was done for these genes in fifteen lung (SCC) and fifteen kidney (RCC) tumours using qPCR. In the majority of the tumour samples mRNA level of both genes was significantly decreased (Figure 5), sometimes more than 50-fold. The difference in expression level in tumour and normal tissues for both SCC and RCC samples was statistically valid (P<0.02). Interestingly, the expression of both genes frequently declined in the same SCC samples. The correlation coefficient according to Spearmen's rank test between *HYAL1* and *HYAL2* mRNA level was 0.67 (P = 0.005) in lung cancer samples (i.e statistically valid). However in RCC samples it was only 0.4 (P = 0.14, i.e not statistically valid). In any case, the results indicated that these genes may play important role in the development of lung and renal malignancies.

What genes could be responsible for the suppression of tumour growth *in vivo*, without any inhibition of cell growth *in vitro*?

There are at least three conceivable categories: genes encoding products required for responding to differentiation inducing signals *in vivo*; products required for normal cellular responses to microenvironmental controls; or genes whose products inhibit angiogenesis [29].

Hyaluronidases are known to play an important role in tumour/host interactions. They may be examples of genes encoding interactive molecules that participate in microenvironmental growth control and carcinogenesis.

There may be many other, as yet unidentified suppressor genes with similarly asymmetric inhibitory properties. The large numbers of LOHs that occur in the major human tumours and are not accounted for by known tumour suppressor genes, as well as the existence of “tumour suppressor gene clusters”, as documented on human chromosome 3p [11], point to a fertile area of further investigation ahead.

## Materials and Methods

### Cell lines, tumour samples and general methods

Paired tumour/normal samples were obtained from Blokhin Cancer Research Center, Russian Academy of Medical Sciences. Altogether fifteen specimens of lung squamous cell carcinoma (SCC) and fifteen clear cell renal carcinoma (RCC) cases and adjacent morphologically normal tissues (conventional “normal” tissues) were obtained from patients after surgical resection of primary tumours and and stored in liquid nitrogen.. Top and bottom sections (3–5 µm thick) cut from frozen tumour tissues were examined histologically and only samples containing 70% or more tumour cells were used in the study. The samples were collected in accordance to the guidelines issued by the Ethic Committee of Blokhin Cancer Research Center, Russian Academy of Medical Sciences (Moscow). All patients gave written informed consent that is available upon request. The study was done in accordance with the principles outlined in the Declaration of Helsinki. All tumor specimens were characterized according to the International System of Clinico-Morphological Classification of Tumors (TNM).

SCLC (small cell lung carcinoma) cell line U2020 was described earlier [30] and RCC (renal cell carcinoma) cell line KRC/Y was obtained from the MTC-KI (Stockholm, Sweden) cell lines collection [1].

Molecular cloning of the human *HYAL1, HYAL2* and *FUS1* genes was described previously [6]. Spontaneous mutant *FUS1mut* containing Val33Met amino acid substitution was isolated from KRC/Y cell line transformed with wild type *FUS1* (unpublished data). These genes were re-cloned into the pETE vector [21]. Recombinants were confirmed by sequencing on ABI310 Sequencer (Applied Biosystems, Foster City, USA) according to manufacturer's protocol.

All molecular, cell biology and microbiology procedures were performed as described previously [21], [31].

Construction of U2020 and KRC/Y cell lines producing tetracycline trans-activator tTA was described in [21].

### Transfection, positive clone selection and Northern blotting

Plasmid DNAs containing *HYAL1, HYAL2, FUS1* and *FUS1mut* genes were purified using R.E.A.L. Prep kit (Qiagen, Valencia, CA). Transfections were performed using Lipofectamine PLUS Reagent (Life Technologies, Rockville, MD) according to the manufacturer's protocol.

After transfection, cells were selected with 200U/ml Hygromycin and 200ng/ml doxycycline for four weeks.

PCR positive clones from each recombinant were grown in Iscove's cell culture medium supplemented with 10% heat-inactivated FBS, 100 µg/ml penicillin, 100 µg/ml streptomycin, 200 U/ml hygromycin and 200ng/ml doxycycline. Each clone was split into two parallel flasks, in one of them cells were grown without doxycycline. After one week, 10^6^ cells were collected and total RNA was isolated with Trizol® reagent (Life Technologies, Rockville, MD).

Northern blotting and hybridization were performed as described before [9], [14], [15]. *HYAL1* and *HYAL2* probes were purified using electrophoresis and the Jetquick Gel Purification kit (Saveen, Germany). The probes were labeled with α-P^32^ dCTP by random labeling.

All growth inhibition experiments *in vivo* and *in vitro* were done as described previously [9], [13], [14], [21]. Work with SCID mice has been approved by North Stockholm Ethical Committee, decision No. 150/08.

Briefly, U2020 or KRC/Y cells were transfected with plasmid DNA using Lipofectamine/Plus reagent (Invitrogen, Germany). Transfected cells were stripped and plated on 100 mm cell culture plates. After selection with 400 µg/ml Hygromycin for 2 weeks, Giemsa-stained colonies were photographed and counted.

The tumorigenicity of each cell line was tested by subcutaneous injection. In total, 5×10^6^ cells were injected into 4-week old female SCID mice. Each mouse received only 1 injection. Twelve control mice were injected with empty pETE vector (six mice were drinking water with DOX and six without). Twenty two mice were used for the experiments (see Table 1). Tumour growth in animals was checked twice a week, if tumour formation was observed, tumours were measured using calipers.

### Cell proliferation assay

Cell proliferation rate was determined using CyQUANT NF Cell Proliferation Assay (Invitrogen) according to the manufacturer's protocol. Briefly, cells were plated at density of 100–500 cells per well in a 96-well plate (totally 8–12 identical wells). Number of cells in wells was counted every 24 hours: growth medium was removed, 50 µl of green-fluorescent CyQUANT GR dye (which exhibits strong fluorescence enhancement when bound to cellular nucleic acid) was added to the well and incubated for 30 min at 37°C. The fluorescence intensity of each sample was measured using a fluorescence microplate reader with excitation at 485nm and emission detection at 530nm. Plotted data points represent averages of triplicate samples, the plotted line is a linear regression fit of all data points. The assay is designed to produce a linear analytical response from at least 100–20,000 cells per well in most cell lines.

### Quantitative real-time PCR (qPCR)

RNA isolated from primary tumour samples was reverse transcribed using the GeneAmp® RNA PCR Kit (Applied Biosystems) according to the manufacturer's protocol. The relative mRNA level of target genes and *GAPDH* was assessed using qPCR (ABI PRISM ® 7000 Sequence Detection System, Applied Biosystems). Ratio of mRNA level of target genes in all analysed tumours was measured relative to the reference “normal” samples (paired morphologically normal tissues obtained from the same patient).

The primers and probes for transcripts were as follows:

*HYAL1:*

Forward primer, 5′- TTTCTGCCCCTGGATGAGC-3′;

Reverse primer, 5′-CTCACCCAGAGCACCACTCC-3′;

Probe, 5′-FAM-CCCAGGCTGTGCTCCAGCTCA-[RTQ1]-3′;

(amplicon size 80 bp);

*HYAL2:*

Forward primer, 5′- CACCACAAGCACGGAGACCT-3′;

Reverse primer, 5′-CAGGCACTAGGCGGAAACTG-3′;

Probe 5′-FAM-CCTTCCTGCATCTCAGCACCAACAG-[RTQ1]-3′;

(amplicon size 197 bp);

*GAPDH:*

Forward primer, 5′- CGGAGTCAACGGATTTGGTC– 3′;

Reverse primer, 5′- TGGGTGGAATCATATTGGAACAT– 3′;

Probe, 5′-FAM-CCCTTCATTGACCTCAACTACATGGTTTACAT–[RTQ1]-3′;

(amplicon size 141 bp);

The optimal primer and probe concentrations for the target and control genes were as follows:

*HYAL1* primers, 300 nM, probe, 300 nM (for lung cancer cDNA samples); primers, 200 nM, probe, 200 nM (for RCC cDNA samples);

*HYAL2* primers, 300 nM, probe, 300 nM (for lung cancer cDNA samples); primers, 200 nM, probe, 200 nM (for RCC cDNA samples);

*GAPDH* primers, 300 nM; probe, 150 nM.

The thermocycler conditions were 10 min at 95°C, then 50 two-step cycles 15sec at 95°C and 60sec at 60° C. qPCR amplification was carried out in triplicate in 25-µl reaction volume using TaqMan Universal Master Mix (Applied Biosystems) and 10ng template cDNA. The sequences of the amplicons were verified by sequencing in 3730 DNA Analyzer automated sequencer (Applied Biosystems).

The comparative C_T_ method was used as described previously [4], [5].

### Statistical analysis

Nonparametric Wilcoxon test was used to compare mRNA expression differences of *HYAL1* and *HYAL2* and reference gene for the same SCC and RCC patients. The evaluation of statistical significance of mRNA level was tested for all studied cases. Nonparametric Spearmen's rank test was used to calculate the coefficient of correlation between the level of mRNA decrease for *HYAL1* and *HYAL2* genes. P-values <0.05 were considered statistically significant. All statistical procedures were performed using BioStat software [32].
